# Expression Profile of Cell Cycle-Related Genes in Human Fibroblasts Exposed Simultaneously to Radiation and Simulated Microgravity

**DOI:** 10.3390/ijms20194791

**Published:** 2019-09-26

**Authors:** Hiroko Ikeda, Masafumi Muratani, Jun Hidema, Megumi Hada, Keigi Fujiwara, Hikaru Souda, Yukari Yoshida, Akihisa Takahashi

**Affiliations:** 1 Gunma University Heavy Ion Medical Center, Maebashi, Gunma 371-8511, Japan; hi-ikeda@gunma-u.ac.jp (H.I.); souda@med.id.yamagata-u.ac.jp (H.S.); yyukari@gunma-u.ac.jp (Y.Y.); 2 Department of Genome Biology, Faculty of Medicine, University of Tsukuba, Tsukuba, Ibaraki 305-8577, Japan; muratani@md.tsukuba.ac.jp; 3 Graduate School of Life Sciences, Tohoku University, Sendai, Miyagi 980-8577, Japan; j-hidema@ige.tohoku.ac.jp; 4 Radiation Institute for Science & Engineering, Prairie View A&M University, Prairie View, TX 77446, USA; mehada@pvamu.edu; 5 Department of Cardiology, University of Texas MD Anderson Cancer Center, Houston, TX 77030, USA; KFujiwara1@mdanderson.org

**Keywords:** simulated microgravity, radiation, combined effects, gene expression, cell cycle

## Abstract

Multiple unique environmental factors such as space radiation and microgravity (μ*G*) pose a serious threat to human gene stability during space travel. Recently, we reported that simultaneous exposure of human fibroblasts to simulated μ*G* and radiation results in more chromosomal aberrations than in cells exposed to radiation alone. However, the mechanisms behind this remain unknown. The purpose of this study was thus to obtain comprehensive data on gene expression using a three-dimensional clinostat synchronized to a carbon (C)-ion or X-ray irradiation system. Human fibroblasts (1BR-hTERT) were maintained under standing or rotating conditions for 3 or 24 h after synchronized C-ion or X-ray irradiation at 1 Gy as part of a total culture time of 2 days. Among 57,773 genes analyzed with RNA sequencing, we focused particularly on the expression of 82 cell cycle-related genes after exposure to the radiation and simulated μ*G*. The expression of cell cycle-suppressing genes (*ABL1* and *CDKN1A*) decreased and that of cell cycle-promoting genes (*CCNB1*, *CCND1*, *KPNA2*, *MCM4, MKI67*, and *STMN1*) increased after C-ion irradiation under μ*G*. The cell may pass through the G_1_/S and G_2_ checkpoints with DNA damage due to the combined effects of C-ions and μ*G*, suggesting that increased genomic instability might occur in space.

## 1. Introduction

Many manned space missions are scheduled in the near future. During such missions, astronauts are continuously exposed to space radiation, which differs from that on Earth. For space missions in low Earth orbit, such as at the International Space Station (ISS), the major source of radiation exposure is solar storms. For exploratory missions beyond low Earth orbit, such as explorations of the Moon and Mars, the effects of exposure to galactic cosmic radiation, including heavy ions, are the most significant health concern. During solar storms, high-dose exposure can have acute effects, including fatigue, nausea, and vomiting [1]. In contrast, during long-duration and exploratory spaceflights, chronic exposure increases the risk of cancer [2,3] and can cause tissue degeneration, development of cataracts [4,5], and potentially affect the central nervous system [6] and immune function [7]. It has also been reported that the risk of cardiovascular disease may be increased by traveling into deep space [8]. However, in another study, this conclusion was questioned because the small number of samples did not enable a statistically robust analysis [9]. Several factors are leading to large uncertainties in the projection of these risks and impeding evaluation of the effectiveness of possible countermeasures; these factors include the type of radiation and the presence of microgravity (μ*G*) [10]. For the assessment and management of human health risks in space, it is necessary to obtain more basic data on the combined effects of radiation under μ*G*.

In previous space experiment, there was no appreciable difference in results between space and ground samples because the time spent in space was short and samples were thus exposed to space radiation at a low dose [11]. In other studies, various organisms have been irradiated before space flight to test the effect of μ*G* on the repair of radiation-induced DNA damage, but again there was no appreciable difference in results [12,13,14,15]. It has been reported that the presence of μ*G* enhances the effects of space radiation [16,17,18], while another study reports improved recovery from radiation damage under μ*G* [19]. Control experiments performed in space under conditions equivalent to Earth’s gravity (1*G*) are limited. The combined effects of μ*G* and radiation thus remain unclear [20,21,22], although it is thought that μ*G* influences the effects of radiation on living organisms. 

In previous ground studies on the combined effects of radiation and μ*G,* a three-dimensional (3D) clinostat [23] or a rotating wall vessel [24] was used to simulate μ*G*, and in order to irradiate samples on these μ*G* simulators, it was necessary to stop rotation during irradiation. As another system to simulate space conditions on the ground, chronic irradiation of samples on the 3D clinostat with neutrons of several MeV from the radioisotope ^252^Cf was reported [25,26], but the effects of radiation were not compared with the status of 1*G* standing samples. Producing actual μ*G* on the ground has limitations in the duration of μ*G* applied when employing the methods such as a drop tower or parabolic flight. It is too short for plants or cultured cells to exhibit obvious changes in growth and development in such method [27]. We thus selected a 3D clinostat for use, which creates simulated μ*G* conditions without time restriction.

Recently, we overcame these previous problems (i.e., discontinuous μ*G* conditions, lack of a 1*G* control experiment) and carried out irradiation experiments under chronic simulated μ*G* conditions. Our 3D clinostat can manipulate the effect of gravity through 3D rotation about two orthogonal axes and through continuously (not randomly) changing the orientation of cells relative to the direction of gravity. This system exerts simulated μ*G* on cells in the direction of gravity on average via mechanical regulation [27,28,29,30]. In parallel with this experimental condition, we performed the same irradiation under 1*G* standing conditions. Using our newly developed simulated μ*G*/irradiation system, we have reported that simultaneous exposure of human fibroblasts to simulated μ*G* and radiation results in more chromosome aberrations than in cells exposed to radiation alone [31]. We know that defects in a cell cycle checkpoint may be responsible for genomic instability [32]. Genes specifically involved in the cell cycle are regulated transcriptionally [33] and are expressed just before they are needed [34]. Therefore, we focused here on the expression of cell cycle-related genes. According to previous reports, the total dose in a mission to Mars may exceed 1 Sv. The maximum allowable effective dose over an astronaut’s lifetime is also around 1 Sv, a level that has been established by several space agencies [35]. Against this background, we irradiated cells with 1 Gy of X-rays or carbon (C)-ions under simulated μ*G*. To address the cause of the combined effects of radiation and simulated μ*G* on genomic instability, we obtained transcriptomic data by RNA sequencing (RNA-seq) in human fibroblasts exposed simultaneously to X-rays or C-ions under simulated μ*G*.

## 2. Results

### 2.1. Gene Expression Profile Changes after Radiation and/or Simulated μG Treatment 

To investigate the profile of genes whose expression changes significantly with simulated μ*G* or radiation treatment alone, screening of genes was carried out using Empirical Analysis of DGE [EDGE, CLC Main Workbench (Qiagen Bioinformatics, Aarhus, Denmark), *p*-value < 0.05, fold change absolute value > 2.0] for each combination of a total of 57,773 genes from RNA-seq analysis in 1BR-hTERT human fibroblasts.

First, to identify genes whose expression levels are altered by simulated μ*G* alone, we compared the expression profile of cells cultured at 1*G* with that of cells exposed to simulated μ*G* for 48 h and found that 140 genes were up-regulated and 103 genes down-regulated. In the pathway analysis using the DAVID Bioinformatics Resources 6.8 (NIAID/NIH, Bethesda, MD, USA) [36] and KEGG (Kanehisa laboratories of Kyoto University, Uji, Kyoto, Japan) databases [37], we found that simulated μ*G* up-regulated a set of genes related to morphine addiction was associated with the gene group for which significant expression changes were observed (Table 1a).

In contrast, for the group of genes that were down-regulated after simulated μ*G* treatment alone, no specific associated pathways were identified. By focusing on the Molecular Function-Direct of Gene Ontology in DAVID [36], we have found that the gene groups showed a tendency for decreased expression were related to calcium ion binding, G-protein coupled purinergic nucleotide receptor activity, and myosin binding (Table 1b). In X-ray or C-ion treatment alone, the major pathways of up-regulated genes (*p53* signaling pathway) and down-regulated genes (cell cycle pathway) tended to be similar. We focused on cell cycle-related genes for further data analysis because the *p53* signaling pathway is also related to the cell cycle.

### 2.2. Radiation Exposure Led to a Marked Change in the Cell Cycle-Related Gene Expression Profile

**Figure 1:**
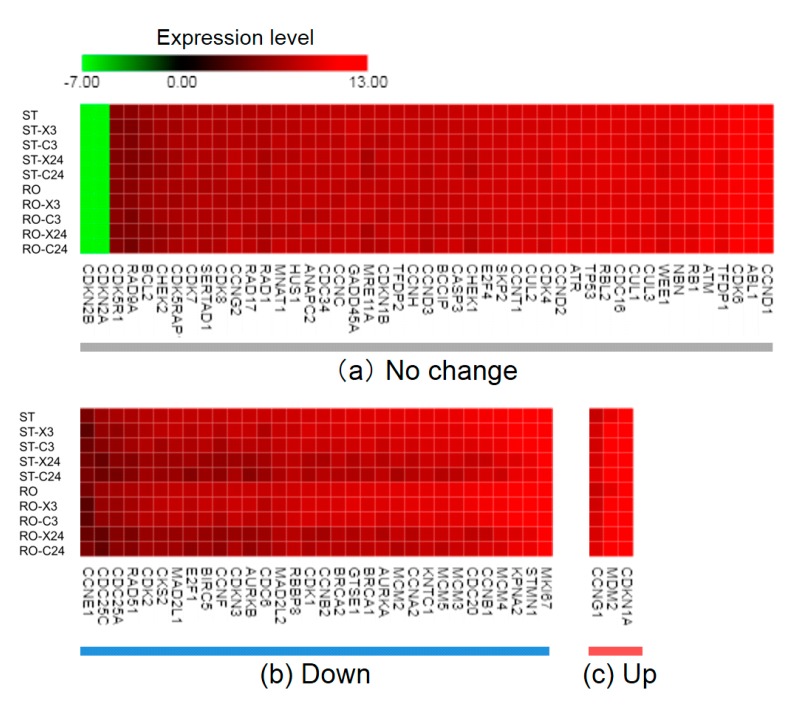
Heatmap of 82 cell cycle-related genes under various conditions. ST, standing 1*G*; RO, rotation for simulated μ*G*; X, X-ray irradiation; C, carbon-ion irradiation; 3, 3 h after irradiation; 24, 24 h after irradiation. After treatment, a total of 36 samples was analyzed for each condition (ST or RO alone, *N* = 6; the other 8 conditions, *N* = 3). The range of expression levels was from −7.00 (yellow-green, down-regulation) to 13.00 (red, up-regulation); black represents 0.00. When the difference between the transformed expression value of a gene under each condition and that of ST was smaller than 1.0, the gene was allocated to the no change group (**a**). If the difference was larger than 1.0, it was put into either the down-regulated (**b**) or up-regulated group (**c**).

On the basis of our RNA-seq results from cells exposed to radiation alone (Table 1), we performed further investigation to identify significant genes related to the cell cycle. Specifically, 84 genes encoding key molecules involved in the cell cycle were selected with information of human cell cycle RT² Profiler PCR Array (Qiagen, Hilden, Germany) [38]. Of those genes, 82 were selected in our sample after RNA-seq. Gene expression profile changes under each condition compared with that in the non-irradiated 1*G* sample are shown as a heatmap in Figure 1. The heatmap represents the extent of gene expression in cells harvested 3 and 24 h after X-ray or C-ion irradiation under simulated μ*G* compared with the expression level of the same gene in non-irradiated samples under 1*G*. The expression levels of up-regulated genes are shown in red and those of down-regulated genes are in green. The up- or down-regulation of gene expression caused by X-ray or C-ion irradiation was more remarkable than the influence of simulated μ*G* (Figure 1b,c) because the pattern did not change markedly between the 1*G* and simulated μ*G* conditions. Upon comparison to the non-irradiated 1*G* sample, the group of genes down-regulated upon radiation exposure alone was related to several key functions for promoting the cell cycle (Figure 1b). In contrast, those up-regulated genes were related to *p53* signaling, such as *CDKN1A* (Figure 1c).

### 2.3. Changes in Cell Cycle-Related Genes Expression Profile in Cells under Simulated μG and Radiation

**Figure 2:**
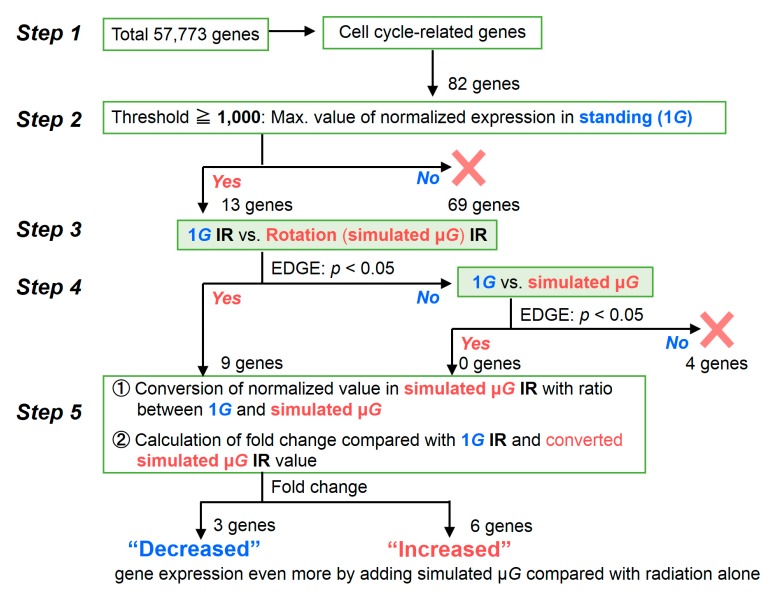
Criteria for identifying genes whose expression levels are different in 1BR-hTERT human fibroblasts when they are exposed simultaneously to radiation and simulated μ*G* vs. when exposed to radiation alone. To narrow down the number of target genes from the total of 57,773 genes, cell cycle-related genes were selected in Step 1. In Step 2, the threshold was set as when the maximum normalized expression value of standing (1*G*) condition was 1000 or more. The normalized expression values of each condition were calculated by CLC Main Workbench software. If this threshold was exceeded, Step 3 involved comparing the simulated μ*G* effect between irradiated samples: normalized expression value of irradiated standing 1*G* samples vs. simulated μ*G* irradiated samples. If Step 3 showed a significant difference (EDGE; *p* < 0.05) between without and with simulated μ*G*, the analysis proceeded to Step 5. If no difference was noted in Step 3, these genes proceeded to Step 4 for judging the simulated μ*G* effect alone: comparison of expression values of 1*G* non-irradiated samples vs. rotation simulated μ*G* condition non-irradiated samples. When genes passed this step (EDGE; *p* < 0.05), they were sent to Step 5 for the calculation of fold change while considering the simulated μ*G* effect alone.

To determine whether the radiation response was enhanced or suppressed by the combination with simulated μ*G*, we focused on genes whose expression specifically changed under the combined conditions. Figure 2 shows the judgement criteria for assessing gene expression profile changes. Of the 57,773 genes targeted for RNAseq analysis, 82 cell cycle-related genes were narrowed down in Step 1. As Step 2, the threshold value was set with the maximum value (> 1000) of the normalized expression in 1*G* sample. Thirteen genes met this criterion in Step 2. Next, the genes that showed significant differences under the influence of radiation were further selected based on the difference in their expression under 1*G* vs. those in simulated μ*G* environment (EDGE; *p* < 0.05) as Step 3. Here, nine out of 13 genes showed significant differences. On the four genes (*ATM*, *CDC20*, *CDK6*, and *TFDP1*) that did not show significant differences, we performed comparative studies between 1*G* non-irradiated cells and simulated μ*G* non-irradiated cells, and examined whether simulated μ*G* by itself had a significant effect. We found that these four genes were not affected by changes in gravitational conditions (Step 4). In Step 5, the expression value in the simulated μ*G* irradiated sample was first converted using the ratio of 1*G* non-irradiated samples vs. simulated μ*G* non-irradiated samples. Next, we calculated the fold change compared with the expression value of 1*G* irradiated samples and the converted expression value of simulated μ*G*-irradiated samples. If the fold change showed a decrease or increase, this means gene expression even more by adding to simulated μ*G* compared with radiation alone.

Nine genes (*ABL1* [39], *CCNB1* [40], *CCND1* [41], *CDKN1A* [40,41], *KPNA2* [42], *MCM4* [43], *MDM2* [44], *MKI67* [45], and *STMN1* [46]) were calculated by considering the effect of simulated μ*G* alone with several processes of Step 5 in judgement criteria (Figure 2). Table 2 provides summary information on the nine selected genes. We could obtain the profile of genes with a change in expression upon adding simulated μ*G* and comparing the results with those obtained with radiation alone. 

**Figure 3:**
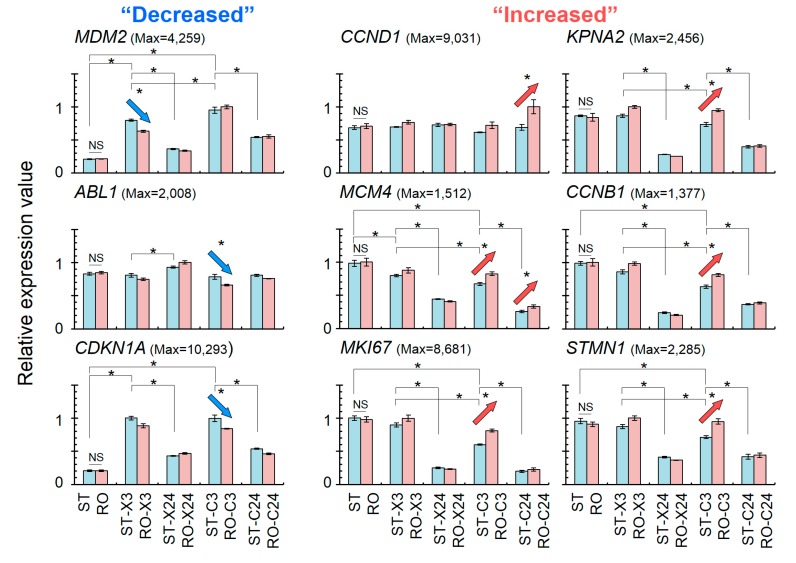
Comparison of relative expression value changes of nine cell cycle-related genes screened via Step 3 and Step 4 in Figure 2; relative expression values were determined by dividing the normalized expression value of each condition by the maximum value (Max) in each gene. The normalized expression values were calculated by CLC Main Workbench software. ST, standing 1*G*; RO, rotation for simulated μ*G*; X, X-ray irradiation; C, carbon-ion irradiation; 3, 3 h after irradiation; 24, 24 h after irradiation. Significantly decreased relative expression values as revealed by statistical analysis with EDGE (* *p* < 0.05, NS = not significant) are shown with blue arrows and increased ones with red arrows. Bar graphs indicate relative expression value ± standard error under each condition (ST or RO alone, *N* = 6; the other 8 conditions, *N* = 3).

The relative expression values of nine cell cycle-related genes were compared among different gravity and radiation conditions, as shown in Figure 3. Simulated μ*G* alone did not have a significant effect on the expression of all nine genes. As a result of X-ray or C-ion exposure alone regardless of the timing of this irradiation, an increased relative expression value was observed for *MDM2*. In addition, X-ray and C-ion irradiation showed no change in *Abl1* but a significant increase in *CDKN1A*, and *CCND1* working downstream of these kinases showed no change in relative gene expression. Moreover, *CCNB1* showed a tendency for a decrease in its relative expression value. There was also a tendency for decreases in the relative expression values of cell cycle-promoting genes such as *KPNA2*, *MCM4*, *MK167*, and *STMN1*. When the same physical dose of 1 Gy of X-rays or C-ions was used in this study, we found that the changes in relative expression value were significantly larger for the C-ion-irradiated samples than for the X-ray-irradiated ones (Figure 3; ST-X3 vs. ST-C3 in *MDM2*, *KPNA2*, *MCM4*, *CCNB1*, *MKI67*, and *STMN1*). The combined treatment gave significantly different results compared with radiation treatment alone in the gene expression of cell cycle checkpoints and promoting proteins.

## 3. Discussion

### 3.1. Gene Expression Profile Changes with Simulated μG

In simulated μ*G* experiments on the ground using a simulator such as a clinostat or a rotating wall vessel, concerns have been raised about the possibility of cells being subjected to shear stress. When attempting to accurately evaluate the effects of simulated μ*G*, it is important to carefully consider the experimental conditions [47] because research groups perform simulated μ*G* experiments under various conditions with different types of simulator and cell line. Todd previously calculated the shear stress on monolayer neuromuscular synapses after rotation, and found it to be almost negligible in the absence of air bubbles in medium with slow rotation [48]. Although the adherent human fibroblasts 1BR-hTERT were used in this study, we completely filled a thin cell culture chamber with medium (without bubbles) to eliminate shear stress on the cells or minimize it as much as possible, based on the report by Todd [48]. It is not necessary to change the medium under our simulated μ*G* conditions until the sampling. From our previous data on cellular growth, which showed no significant difference between rotating and standing conditions after 48 h of culture [29], it is unlikely that cells are subjected to shear stress under our experimental conditions using our system.

### 3.2. X-ray and C-Ion Irradiation Induced Changes in Gene Expression

From the results in Table 1, major cellular pathways targeted by X-ray and C-ion irradiation were up-regulated *p53* signaling and down-regulated cell cycle which work downstream the *p53* signaling pathway. The heatmap focused on genes involved in the cell cycle not only revealed similar results to those shown in Table 1, but also confirmed that the effect of radiation alone was greater than that of simulated μ*G* treatment alone (Figure 1). These results were suggested to be consistent with the typical radiation-induced cell cycle checkpoints and subsequent repair responses reported so far [49,50]. The relative expression value change due to radiation alone of the cell cycle-related genes selected according to the judging criteria is shown in Figure 3. The expression of *ABL1* did not change, while *CDKN1A* also known as *p21* was up-regulated. *CCND1* related to Cyclin D did not show a change of expression downstream of *ABL1* and *CDKN1A* at both 3 and 24 h after irradiation. *p21* expression increased upon C-ion irradiation, which matches the finding in a previous report [51]. 

Moreover, the expression level of *KPNA2* was decreased and that of *CDKN1A* was increased, while the level of *CCNB1* located downstream of them was decreased. *MCM4*, *MKI67*, and *STMN1*, which promote the cell cycle, showed a tendency to be down-regulated (Table 2). These results suggest that 1BR-hTERT has normal cell cycle checkpoints, including not only G_1_ arrest but also G_2_ arrest. 

### 3.3. Synergistic Effect of Radiation and Simulated μG on Changes in Expression Profile of Cell Cycle-Related Genes

To simulate space conditions of radiation and μ*G* with our system, samples were pulse-irradiated every minute. It has been estimated that cells are actually subjected to space radiation of high linear energy transfer (LET) particles (a few times per day) in space [52]. As a limitation of our system, the dose rate of space radiation during space flight is lower than 0.03 Gy/min as used in this study. However, our system enables irradiation without stopping rotation, and it has the unique advantage of simulating space conditions by comparison with the effects of X-rays and C-ions on the ground. 

The results of several experiments focused on the cell cycle under simulated μ*G* have been reported using various cell lines. For example, simulated μ*G* induced partial G_1_ phase arrest in rat pheochromocytoma PC12 cells [53]. In addition, both normal murine vascular smooth muscle cells and neoplastic human breast cancer cells were induced to undergo partial arrest at G_2_/M and showed increased expression of *CDKN1A* upon simulated μ*G* [54]. Moreover, in murine microvascular endothelial 1G11 cells, cell growth was inhibited and *p21* was induced by simulated μ*G* [55]. 

In contrast, Arase et al. reported that simulated μ*G* reduced the expression of *p21* in human fibroblasts [56]. Although the adaptation and responses to μ*G* may differ depending on the cell type, target factors, and the treatment time [54], these previous reports suggest that μ*G* is an important factor regulating the cell cycle through the *p53* signaling pathway.

In this study, we found that a total of 140 genes were up-regulated and 103 genes were down-regulated by simulated μ*G* treatment alone. A small group of up-regulated genes was associated with morphine addiction-related pathways, but no major pathways were identified for the down-regulated genes. The use of the Molecular Function-Direct by Gene Ontology with DAVID also revealed that some down-regulated genes were associated with processes related to the muscle contraction such as *ACTA1*. Similar result have previously been reported [25], thus our findings confirmed that our simulated μ*G* system functions appropriately and that it is an effective tool for further investigation of the combined effect of radiation and simulated μ*G*.

Our results after combined treatment with C-ion irradiation and simulated μ*G* revealed synergistic changes in the expression of genes (Figure 3). The expression of *CDKN1A* also known as *p21* was decreased at 3 h and that of *CCND1* was increased at 24 h after the treatment through down-regulating *ABL1* (3 h) and leaving *TP53* unchanged. Therefore, the results suggest that G_1_ arrest does not occur under combined conditions of C-ion irradiation and simulated μ*G*. Moreover, *KPNA2* and *CCNB1* were up-regulated with a decrease of *CDKN1A* at 3 h after the treatment, and then G_2_ arrest may not occur. Based on previous reports, our results suggest that C-ion irradiation alone may induce cell cycle checkpoints normally, but the checkpoints are released by adding simulated μ*G* treatment. 

While radiation treatment alone tended to suppress the cell cycle (Figure 3), the combined effect of C-ion irradiation and simulated μ*G* may promote cell proliferation. Indeed, previous report show that simulated μ*G* promotes the proliferation and differentiation of human mesenchymal stem cells [57]. Similar findings were also made in experiments using human dental pulp stem cells [58] and human epidermal stem cells [59], including the result of an increased percentage of Ki67-positive cells. These reports support our finding that C-ion irradiation and simulated μ*G* together promote cell cycle progression.

**Figure 4:**
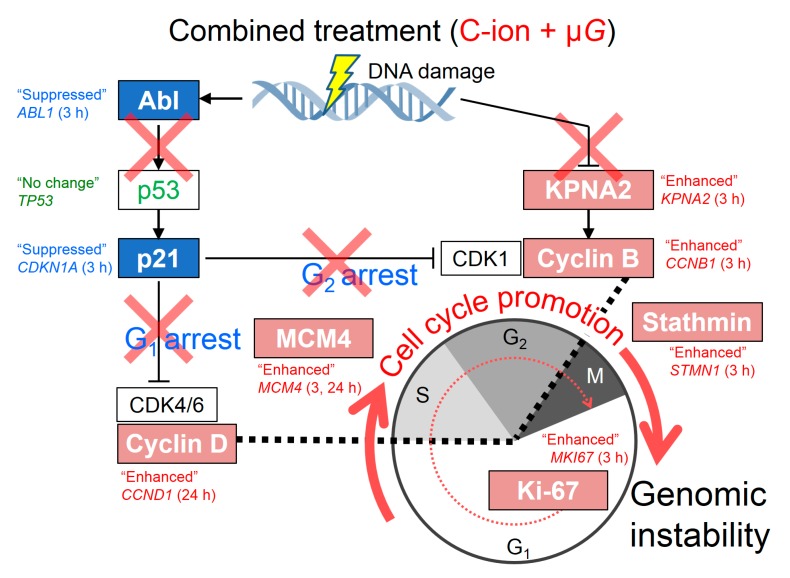
Schematic representation of the proposed model of cell cycle-related pathway modified by combined effect of C-ion exposure with simulated μ*G* in 1BR-hTERT human fibroblasts. The pink column shows cell cycle-promoting genes and blue column indicates cell cycle-suppressing ones.

On the basis of our results (Figure 3), we propose a model by which the cell cycle-related pathway is modified by the combined effect of C-ion exposure and simulated μ*G* in 1BR-hTERT human fibroblasts (Figure 4). Cells may pass through each cell cycle checkpoint with DNA damage after combined treatment with C-ion irradiation and simulated μ*G*. We reported that combined treatment of cells with simulated μ*G* and radiation induced a higher frequency of both simple and complex types of chromosome aberrations compared with the level in cells irradiated with X-rays or C-ions alone under the 1*G* standing condition [31]. This proposed model for the modified cell cycle pathway may provide some insights into the mechanism for increased chromosome aberration due to the combined effect of C-ion and simulated μ*G*.

In terms of the effect on 1BR-hTERT fibroblasts of X-ray irradiation in combination with simulated μ*G*, only one gene, *MDM2*, showed a significant decrease in its expression. Although *MDM2* is known as one of the components that negatively feeds back to *p53* signaling, its expression was synergistically decreased by the combined effect relative to the effect of X-ray irradiation alone; thus, there is a possibility that accumulation of p53 protein may occur and that the cell cycle checkpoint works downstream of *p53*. Therefore, it is possible that there is no significant difference after combined treatment with X-ray irradiation in this study. Another potential reason for this result is the radiation dose used in this experiment. We used 1 Gy for both C-ions and X-rays. From the cell survival curve [31], a dose equivalent to 1 Gy C-ions would be 2 Gy by X-rays, and therefore a significant difference in the expression of some of the genes may not have been seen in this study with X-rays. However, the changes in gene expression profile due to the combined effect in genes involved in the cell cycle regulation showed a similar tendency between X-ray and C-ion treatments.

Previous studies on simulated μ*G* experiments with radiation at the ground level that support our current model. Combined effects of these factors caused increases in double-strand breaks [60] and genomic instability such as the formation of micronuclei [61], a decrease in cell cycle checkpoints, and enhancements of DNA damage response (by γ-rays) [62] and chromosome aberrations (by X-rays) [63]. In a previous transcriptomic study, normal human bronchial epithelial cells exposed to heavy ions (silicon or iron ions) as high-LET radiation were compared with those exposed to γ-rays as low-LET radiation. The results revealed the distinct expression patterns of genes related to cell cycle regulation, DNA damage response, and other stress-responding mechanisms that were specific to the radiation quality [64]. In space experiments involving simultaneous exposure to space radiation and μ*G*, several different sets of results have been reported, with suppression of the cell cycle through activated *p21* [65] and cell proliferation [66], but also no change in the amount of p21 protein [67,68]. The reason for this difference may be the short stay in space and the lack of a sufficient dose to induce radiation effects. However, DNA damage has been detected [69,70,71] after time spent in space, and increases in genomic instability have also been shown in the NASA Twins Study [72]. In the review article, additional transcriptomic data presented that the pathway of nuclear factor kappa-light-chain-enhancer of activated B cells is well known to be altered by either μ*G* or space radiation. This appeared potentially to have adverse impacts on health [73]. When non-dividing human fibroblasts were studied on the ISS for 14 days, no differences in gene or miRNA expression profiles were found upon comparison with ground samples. However, it has been shown that gene or miRNA expression profiles also change depending on the number of days of culture and the cellular conditions [74].

It is known that some changes in gene expression profile in ground-based experiments using a simulator mimicking μ*G* conditions are not consistent with the changes seen in space experiments [75]. Therefore, it is necessary to compare comprehensively the specific genes screened by simulation experiments on the ground with transcriptomic data obtained in space experiments. Our results, which may indicate the release of checkpoints and promotion of the cell cycle by combined effects of C-ion irradiation and simulated μ*G*, help to shed light on the mechanism behind the findings in these previous reports. The results also show the need to consider combined effects of simultaneously radiation and μ*G* exposure on the risk assessment based on previous dose-response data obtained from irradiated cells under 1*G* conditions. 

## 4. Materials and Methods 

### 4.1. Cell Culture

Human fibroblasts (1BR-hTERT cells) were kindly provided by Dr. P.A. Jeggo (University of Sussex, Brighton, UK) and Dr. A. Shibata (Gunma University Initiative for Advanced Research (GIAR), Maebashi, Gunma, Japan). Cells were cultured in CO_2_-independent medium (Thermo Fisher Scientific, Waltham, MA, USA) supplemented with 10% (*v*/*v*) fetal bovine serum (MP Biomedicals, Santa Ana, CA, USA), 200 mM l-glutamine (Thermo Fisher Scientific), and penicillin–streptomycin mixed solution (Nacalai Tesque, Kyoto, Kyoto, Japan) at 37 °C. Exponentially growing cells were cultured in disposable sealed irradiation cell culture chambers (DCC) (Chiyoda Co., Yokohama, Kanagawa, Japan) [76,77] that were completely filled with fresh medium (without bubbles) before setting in the 3D clinostat [PMS-CST I; Advanced Engineering Services Co. Ltd. (AES), Tsukuba, Ibaraki, Japan] for simulated μ*G* or a static stage (AES) as a 1*G* control, as previously reported [29]. 

### 4.2. Synchronized Irradiation Systems Under Simulated μG or 1G

**Figure 5:**
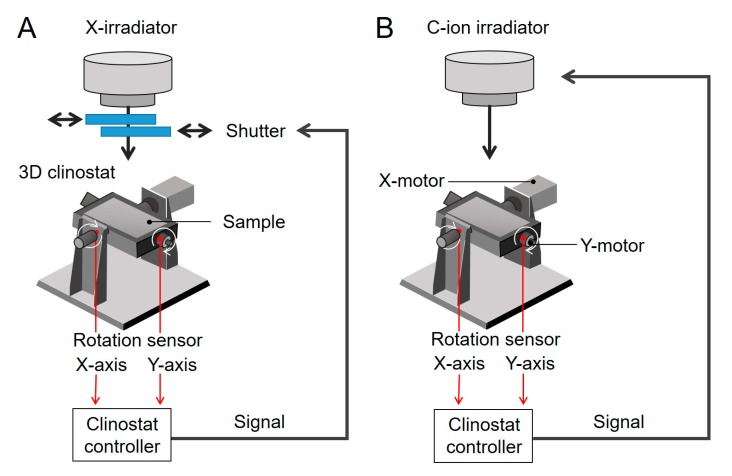
Schema of 3D clinostat synchronized irradiation systems for studying the combined effects of radiation and simulated μ*G*. For synchronization, X-ray irradiation using a high-speed shutter (**A**) and C-ion irradiation with a respiratory gating system as used in heavy ion radiotherapy were performed (**B**).

Irradiation of cells without stopping clinostat motion was achieved by 0.2 sec of pulse irradiation when the cell growth surface of the chamber on the clinostat became perpendicular to the beam of irradiation. The controller of the 3D clinostat was also connected to a high-speed shutter system (Accelerator Engineering Co. (AEC), Chiba, Chiba, Japan) for X-ray irradiation (Figure 5A) or a respiratory gating system for C-ion irradiation (Figure 5B) to achieve this specific positioning (i.e., synchronization) of the chamber orientation and the timing of the pulse irradiation, which occurred every 60 sec. The X:Y ratios of clino-rotation were set at 11:13 rpm and = 66°/s:78°/s to accurately synchronize irradiation when the samples were in a horizontal position [28,29]. 

Synchronized X-ray irradiation was performed using an X-ray generator [200 kV, 14.6 mA, aluminum filter (0.3 mm thick), MultiRad225; Faxitron Bioptics, LLC, Tucson, AZ, USA] equipped with a high-speed shutter. Synchronized C-ion irradiation was performed using a synchrotron (Gunma University Heavy Ion Medical Center (GHMC), Maebashi, Gunma, Japan) and respiratory gating signals with a dose-averaged LET of 50 keV/μm at the center of the 6-cm spread-out Bragg peak of the beam with energy of 290 MeV/n [78]. As a control, cells in the same chamber mounted on a stationary clinostat (1*G*) were pulse-irradiated for 0.2 sec every 60 sec [28,29]. The dose used was 1 Gy of X-rays or C-ions, and the dose rate was approximately 0.03 Gy/min for both X-ray and C-ion irradiation under the simulated μ*G* or 1*G* conditions.

### 4.3. Experimental Design

**Figure 6:**
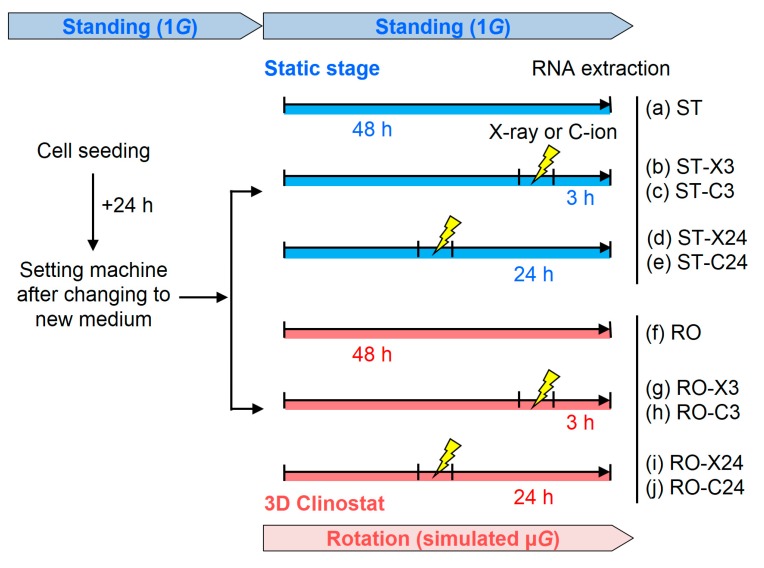
Experimental design from cell seeding to RNA extraction using human fibroblasts. ST (**a**–**e**) and RO conditions (**f**–**j**). No irradiation (**a**,**f**), X-ray (**b**,**d**,**g**,**i**), or C-ion (**c**,**e**,**h**,**j**) irradiation.

Comprehensive gene expression analysis of human fibroblasts was performed to determine the combined effects of irradiation and simulated μ*G*. 3D clinostat-synchronized X-ray or C-ion irradiation at 1 Gy was performed without stopping rotation. Samples were set on the static stage for standing (ST) 1*G* and the 3D clinostat for rotation (RO) simulated μ*G* after changing to new medium at 24 h after the seeding of cells. The cells were maintained for 3 or 24 h after X-ray or C-ion irradiation as part of total culture time of 2 days in standing or rotating conditions, and then total RNA was isolated from the cells (Figure 6).

### 4.4. RNA Extraction

The DCC samples were continuously cultured under standing or rotating conditions for 3 or 24 h after 1 Gy irradiation with X-rays or C-ions. Immediately after these treatments, adherent cells were lysed in 1.6 mL of TRIzol^®^ Reagent (Thermo Fisher Scientific) for homogenization and frozen at −80 °C. Gene expression was analyzed by Tsukuba i-Laboratory LLP (Tsukuba, Ibaraki, Japan).

### 4.5. RNA Sequencing

A total of 36 samples were analyzed (1*G* or simulated μ*G* alone, *N* = 6; the other 8 conditions, *N* = 3) using RNA sequencing. RNA sequence reads quantified 57,773 genes. After RNA sequencing, the profiles of genes with up- or down-regulation of their expression were listed according to ratios of the expression value using EDGE (*p* < 0.05, fold change with absolute value >2.0) with the CLC Main Workbench software.

### 4.6. Pathway Analysis

To identify cellular pathways within these lists after narrowing down the total of 57,773 genes by statistical analysis using EDGE of the CLC Main Workbench, we used the Functional Annotation Tool in DAVID Bioinformatics Resources 6.8 [36]. Using the KEGG pathway database, we selected the top three pathways which show a higher percentage in narrowing down pathways (*p* < 0.05). If no specific pathway was identified by KEGG tools, we selected the top three functions that show a higher percentage (*p* < 0.05) focusing on the Gene Ontology-Molecular Function in DAVID.

### 4.7. Heatmap Representation for Visualization of Changing Gene Expression Level

The expression values of each condition were normalized using CLC Main Workbench software for the screened group of 82 cell cycle-related genes. After adding 0.01 to normalized expression values, log 2 conversion was performed as transformed values. The gene expression level changes were presented as a heatmap using the transformed values calculated through these multiple steps with CLC Main Workbench software. To create the heatmap, versatile matrix visualization and the analytical software Morpheus (https://software.broadinstitute.org/morpheus) were used. Using a standing 1*G* non-irradiated sample (ST), if the difference of the transformed value of each condition vs. ST was smaller than 1.0, allocation to the no change group was performed (**a**). However, if the difference was larger than 1.0, allocation to the Down-regulated (**b**) or Up-regulated group (**c**) was performed.

### 4.8. Statistical Analysis 

To observe the change in gene expression profile after exposure to the radiation and simulated μ*G*, each sample was analyzed several times (total 36 samples; 1*G* or simulated μ*G* alone, *N* = 6; the other 8 conditions, *N* = 3) at Tsukuba i-Laboratory LLP. The profiles of the up- or down-regulation of gene expression were listed according to the ratios of the expression value using EDGE with CLC Main Workbench software. In all statistical analyses, differences were considered significant at *p*-values less than 0.05. For selecting genes among the total of 57,773 genes, fold changes with an absolute value larger than 2.0 were considered statistically significant with *p*-values of less than 0.05. The bar graph of Figure 3 shows relative expression value ± standard error for each condition (ST or RO alone, *N* = 6; the other 8 conditions, *N* = 3). 

## 5. Conclusions

In this study, we achieved to identify nine cell cycle-related genes that show synergistic changes by combined effects with X-ray or C-ion irradiation under simulated μ*G*. Radiation treatment alone with X-rays or C-ions increased the gene expression of *CDKN1A* (*p21*), while each cell cycle checkpoint continued to work normally. However, the combined effects of C-ions and simulated μ*G* decreased the expression of *CDKN1A*, which may have resulted in failure to achieve arrest at checkpoints; this promoted the cell cycle without sufficiently undergoing steps of DNA damage repair. Simulated μ*G* may be one of the key factors that synergistically change the effect of radiation at ground level, and changes in the expression of cell cycle-related genes indicated the possibility of genomic instability including chromosomal abnormalities. To assess the risk of radiation in future long-term stays in space, further ground and space experiments need to be conducted, taking into consideration the results obtained here.

## Figures and Tables

**Table 1:** Numbers of up- and down-regulated gene sets and the top three of related cellular pathways after simulated μ*G* or radiation treatment in human fibroblasts.

(**a**) Genes up-regulated by radiation and/or simulated μ*G*		
**vs. ST**	**Total Genes**	**Cellular Pathways (Number of Genes)**
ST-X3	315	*p53* signaling (7), FoxO signaling (5), Adrenergic signaling in cardiomyocytes (5)
ST-X24	523	Neuroactive ligand–receptor interaction (13), Calcium signaling (11), cAMP signaling (11)
ST-C3	253	Cytokine–cytokine receptor interaction (7), *p53* signaling (6), Measles (5)
ST-C24	350	*p53* signaling (6), FoxO signaling (5)
RO-X3	204	*p53* signaling (4)
RO-X24	674	Neuroactive ligand–receptor interaction (17), Calcium signaling (11), cAMP signaling (11)
RO-C3	211	*p53* signaling (7), Cytokine–cytokine receptor interaction (6)
RO-C24	339	PI3K–Akt signaling (10), *p53* signaling (6), ABC transporters (5)
RO	140	Morphine addiction (3)
(**b**) Genes down-regulated by radiation and/or simulated μ*G*		
**vs. ST**	**Total Genes**	**Cellular Pathways (Number of Genes)**
ST-X3	79	Cell cycle (5), MicroRNAs in cancer (4), *p53* signaling (3)
ST-X24	439	Systemic lupus erythematosus (46), Alcoholism (46), Cell cycle (32)
ST-C3	198	Cell cycle (5), Systemic lupus erythematosus (4)
ST-C24	663	Systemic lupus erythematosus (55), Alcoholism (55), Cell cycle (36)
RO-X3	86	Pathways in cancer (5)
RO-X24	507	Alcoholism (46), Systemic lupus erythematosus (45), Cell cycle (33)
RO-C3	210	Pathways in cancer (7)
RO-C24	702	Systemic lupus erythematosus (55), Alcoholism (55), Cell cycle (35)
RO	103	* Calcium ion binding (6), * G-protein coupled purinergic nucleotide receptor activity (2), * Myosin binding (2)

ST, standing 1*G*; RO, rotation for simulated μ*G*; X, X-ray irradiation; C, carbon-ion irradiation; 3, 3 h after irradiation; 24, 24 h after irradiation. * The list by Gene Ontology-Molecular Function-Direct with DAVID (*p* < 0.05, top three show a higher percentage in narrowing as biological process).

**Table 2:** Summary of nine cell cycle-related genes affected by combined treatment with radiation and simulated μ*G* identified by the screening criteria shown in Figure 2.

Cell Cycle	Gene ID (Protein)	Ensembl	Function	Ref.
Suppression	*ABL1* (c-Abl)	ENSG00000097007	This gene encodes a protein tyrosine kinase. C-Abl protects p53 by antagonizing the inhibitory effect of Mdm2, an action that requires direct interplay between c-Abl and Mdm2.	[39]
	*CDKN1A* (p21)	ENSG00000124762	The encoded protein binds to and inhibits the activity of cyclin D1–CDK4/6 or cyclin B1–CDK1 complexes, and thus functions as a regulator of cell cycle progression at G_1_ and G_2_.	[40,41]
Promotion	*CCNB1* (Cyclin B1)	ENSG00000134057	Activated cyclin B1 with CDK1 promotes several of the events of early mitosis. DNA damage leads to nuclear accumulation of inactive cyclin B1–CDK1 complexes by p21, and contributes to the establishment of permanent G_2_ arrest.	[40]
	*CCND1* (Cyclin D1)	ENSG00000110092	This cyclin forms a complex with and functions as a regulatory subunit of CDK4/6, whose activity is required for cell cycle G_1_/S transition. DNA damage leads to nuclear accumulation of inactive cyclin D1–CDK4/6 complexes by p21, and contributes to the establishment of G_1_ arrest.	[41]
	*KPNA2* (KPNA2)	ENSG00000182481	KPNA2 expression accelerates cell cycle progression by up-regulating cyclin B and CDK1.	[42]
	*MCM4* (MCM4)	ENSG00000104738	MCM4, a subunit of a putative replicative helicase, is essential for the initiation of eukaryotic genome replication. MCM4 is one of the crucial targets of the DNA replication checkpoint system.	[43]
	*MDM2* (MDM2)	ENSG00000135679	MDM2 can promote tumor formation by targeting tumor suppressor p53 proteins for proteasomal degradation. Mdm2 promotes Cdc25C protein degradation and delays cell cycle progression through the G_2_/M phase.	[44]
	*MKI67* (Ki-67)	ENSG00000148773	Ki-67 is associated with and may be necessary for cellular proliferation. Ki-67 contributes to normal cell cycle progression.	[45]
	*STMN1* (Stathmin 1)	ENSG00000117632	Stathmin 1 is a ubiquitous cytosolic phosphor-protein. Stathmin is critically important not only for the formation of a normal mitotic spindle upon entry into mitosis but also for regulation of the function of the mitotic spindle in the later stages of mitosis and for the timely exit from mitosis.	[46]

## Abbreviations

3D	Three-dimensional
*ABL1*	ABL proto-oncogene 1, non-receptor tyrosine kinase (c-Abl)
*ACTA1*	Actin alpha 1, skeletal muscle
AEC	Accelerator Engineering Co.
AES	Advanced Engineering Services Co. Ltd.
*ATM*	ATM serine/threonine kinase
*CCNB1*	Cyclin B1
*CCND1*	Cyclin D1
*CDC20*	Cell division cycle 20
CDK1	Cyclin-dependent kinase 1 (= cell division cycle protein 2, cdc2)
CDK4/6	Cyclin-dependent kinase 4/6
*CDKN1A*	Cyclin-dependent kinase inhibitor 1A (= p21)
C-ion	Carbon ion
DAVID	Database for Annotation, Visualization, and Integrated Discovery
DCC	Disposable sealed irradiation cell culture chamber
EDGE	Empirical Analysis of DGE
GHMC	Gunma University Heavy Ion Medical Center
GIAR	Gunma University Initiative for Advanced Research
ISS	International Space Station
KEGG	Kyoto Encyclopedia of Genes and Genomes
*KPNA2*	Karyopherin subunit alpha 2
LET	Linear energy transfer
*MCM4*	Minichromosome maintenance complex component 4
*MDM2*	MDM2 proto-oncogene
MeV/n	Megaelectronvolt per nucleon
μ*G*	Microgravity
*MKI67*	Marker of proliferation Ki-67
NIAID	National Institute of Allergy and Infectious Diseases
NIH	National Institutes of Health
p53	p53 tumor suppressor protein
RO	Rotation for simulated μ*G*
ST	Standing 1*G*
*STMN1*	Stathmin 1
*TFDP1*	Transcription factor Dp-1
